# Antibiotic prescribing practices of dentists for endodontic infections; a cross-sectional study

**DOI:** 10.1371/journal.pone.0244585

**Published:** 2020-12-30

**Authors:** Sheela B. Abraham, Nizam Abdulla, Wan Harun Himratul-Aznita, Manal Awad, Lakshman Perera Samaranayake, Hany Mohamed Aly Ahmed

**Affiliations:** 1 Department of Preventive and Restorative Dentistry, College of Dental Medicine, University of Sharjah, Sharjah, United Arab Emirates; 2 Department of Restorative Dentistry, Faculty of Dentistry, University of Malaya, Kuala Lumpur, Malaysia; 3 Department of Oral and Craniofacial Health Sciences, College of Dental Medicine, University of Sharjah, Sharjah, United Arab Emirates; 4 Department of Oral & Craniofacial Sciences, Faculty of Dentistry, University of Malaya, Kuala Lumpur, Malaysia; 5 Faculty of Dentistry, The University of Hong Kong, Hong Kong; Danube Private University, AUSTRIA

## Abstract

**Objective:**

The indiscriminate prescription of antibiotics has led to the emergence of resistance microbes worldwide. This study aimed to investigate the antibiotic prescribing practices amongst general dental practitioners and specialists in managing endodontic infections in the United Arab Emirates (UAE).

**Design:**

General dental practitioners and specialists in the UAE were invited to participate in an online questionnaire survey which included questions on socio-demographics, practitioner’s antibiotic prescribing preferences for various pulpal and periapical diseases, and their choice, in terms of the type, dose and duration of the antibiotic. The link to the survey questionnaire was sent to 250 invited dentists. Data were analyzed by descriptive statistics and chi-square tests for independence and level of significance was set at 0.05.

**Results:**

A total of 174 respondents participated in the survey (response rate = 70%). The respondents who prescribed antibiotics at least once a month were 38.5% while 17.2% did so, more than three times a week; amoxicillin 500 mg was the antibiotic of choice for patients not allergic to penicillin (43.7%), and in cases of penicillin allergies, erythromycin 500 mg (21.3%). There was a significant difference in the antibiotic prescribing practices of GDPs compared to endodontists and other specialties especially in clinical cases such as acute apical abscesses with swelling and moderate to severe pre-operative symptoms and retreatment of endodontic cases (p<0.05). Approximately, three quarters of the respondents (78.7%) did not prescribe a loading dose when prescribing antibiotics. About 15% respondents prescribed antibiotics to their patients if they were not accessible to patients due to a holiday/weekend.

**Conclusions:**

In general, the antibiotic prescribing practices of UAE dentists are congruent with the international norms. However, there were occasions of inappropriate prescriptions such as in patients with irreversible pulpitis, necrotic pulps with no systemic involvement and/or with sinus tracts.

## Introduction

Pulpal inflammations and pain associated with root canal infections generally require an operative intervention for relief of symptoms and not necessarily the prescription of systemic antibiotics [1, 2]. The mandatory use of an antibiotic in general is limited to infections associated with fever, and evidence of systemic spread such as lymphadenopathy and trismus [1]. The American Association of Endodontists (AAE) for instance, recommends antibiotic prescription in medically healthy patients when diffuse swelling is present due to an apical abscess, or in an infection with systemic symptoms such as fever and malaise [1]. Similarly, the European Society of Endodontology (ESE) recommends systemic adjunctive antibiotic therapy in acute apical abscess with associated fever, malaise, lymphadenopathy, trismus and progressive infections such as cellulitis or osteomyelitis [2].

Various surveys in UK and elsewhere, have shown that of the overall antibiotics prescribed by clinicians in general, approximately 10 per cent are due to those written by dentists [3]. A survey of 6000 dentists in UK revealed that almost 40% of dentists prescribed antibiotics at least thrice a week, and 15% on a daily basis [4]. Studies have also shown significant variations amongst antibiotic prescribing habits amongst clinicians in terms of the dose and duration for almost identical infections [5–7].

Due to such indiscriminate use of antibiotics, in general, there has been a rapid emergence of bacterial resistance to common antibiotics, particularly broad-spectrum variants which are popular and over prescribed [8, 9]. In dentistry, recent reports have documented the isolation of resistant bacteria from deep neck infections of odontogenic origins as well as from primary and persistent infections [10, 11]. Also, horizontal transfer of bacterial-resistant genes between different bacterial species within the root canal has been noted by some [12]. The inappropriate antibiotic prescribing habits of dentists have been attributed to factors such as lack of knowledge, patient satisfaction, and related social factors [13, 14].

In view of the foregoing, it is important to assess the antibiotic provision by dentists to patients for endodontic infections, and to reduce their unwarranted consumption [15]. Various workers have analysed the prescription of antibiotics in dentistry either in the university clinic settings [6], or in community settings via community surveys [16], or by evaluating the prescriptions per se [5] A recent study on the knowledge and attitude of dentists to prescribing antibiotics in the northern emirates of United Arab Emirates (UAE) by Al Khabuli et al., has shown that dentists had a fair knowledge of antibiotic use and abuse [16]. However, as far as we are aware, the pattern of prescribing antibiotics to manage endodontic infections in UAE, has not been assessed. Hence the aim of this study was to investigate the antibiotic prescribing practices of dentists in the latter jurisdiction focusing on the management of endodontic infections.

## Materials and methods

This was a prospective, cross-sectional online survey based on a questionnaire previously used by Germack et al. [17], but with minor modifications. The survey was conducted following the approval of the Research Ethics Committee of the University of Sharjah (No REC-18-05-30-01). The review committee waived the need for consent as it was an online survey for dentists and only those who consented participated in the survey.

The survey instrument was first reviewed and validated by two endodontists for clarity, and relevance. Afterwards an online version of the questionnaire was created using Google forms. The link to the survey was send by email to 250 members of the Emirates Dental Society, a branch of the Emirates Medical Association; a non-profit organization of licensed health practitioners in the UAE. These dentists were registered with the Abu Dhabi Health Authority (SEHA), Dubai Health Authority (DHA) or the Ministry of Health (MOH) and were employed in all seven emirates of the UAE [viz, Abu Dhabi, Dubai, Sharjah, Ajman, Um Al-Quwain (UAQ), Ras Al-Khaimah (RAK) and Fujairah].

Based on previous research [18], it was estimated that approximately 13% of the sample prescribe an antibiotic in a situation where antibiotics is not required, using 5% margin of error and a power of 80%, and a 0.05 level of significance (two tailed), a total of 173 participants were needed for the survey.

A list of dentists who were registered in the Emirates Dental Society was obtained, and all those who were actively registered were sent the online survey. Of these, dentists working in the government health centers/ private practices /university clinics in Abu Dhabi, Dubai and Sharjah (60 each), as well as 20 each from Um Al Quwain (UAQ), Ajman and RAK, and 10 from Emirate of Fujairah were invited to participate in the study. An invitation to participate was e-mailed with an explanation of the objectives of the study and instructions on completion of the questionnaire. The link was open for 4 weeks, for a response, and closed thereafter. The questionnaire was formulated to elicit demographic data, practitioner’s antibiotic prescribing preferences in different clinical situations, the type, dose and duration of the antibiotics. (the survey form in S1 Appendix).

### Data analysis

The Statistical Package for Social Sciences (SPSS) Version 26.0 (IBM., Armonk, NY, USA) was used for data processing and analysis. Participant characteristics were described using frequency distribution for categorical variables. Participants’ scope of practice was divided into three categories: General Dental Practitioners (GDPs), Endodontists and Other dental specialties (namely oral surgeons, prosthodontists, orthodontists, periodontists, implant surgeons, etc.), the associations between scope of practice, various clinical situations and frequency of antibiotics prescribing were assessed using Chi-square test (p = 0.05), Bonferonni adjustment were made for multiple testing.

## Results

**Fig 1:**
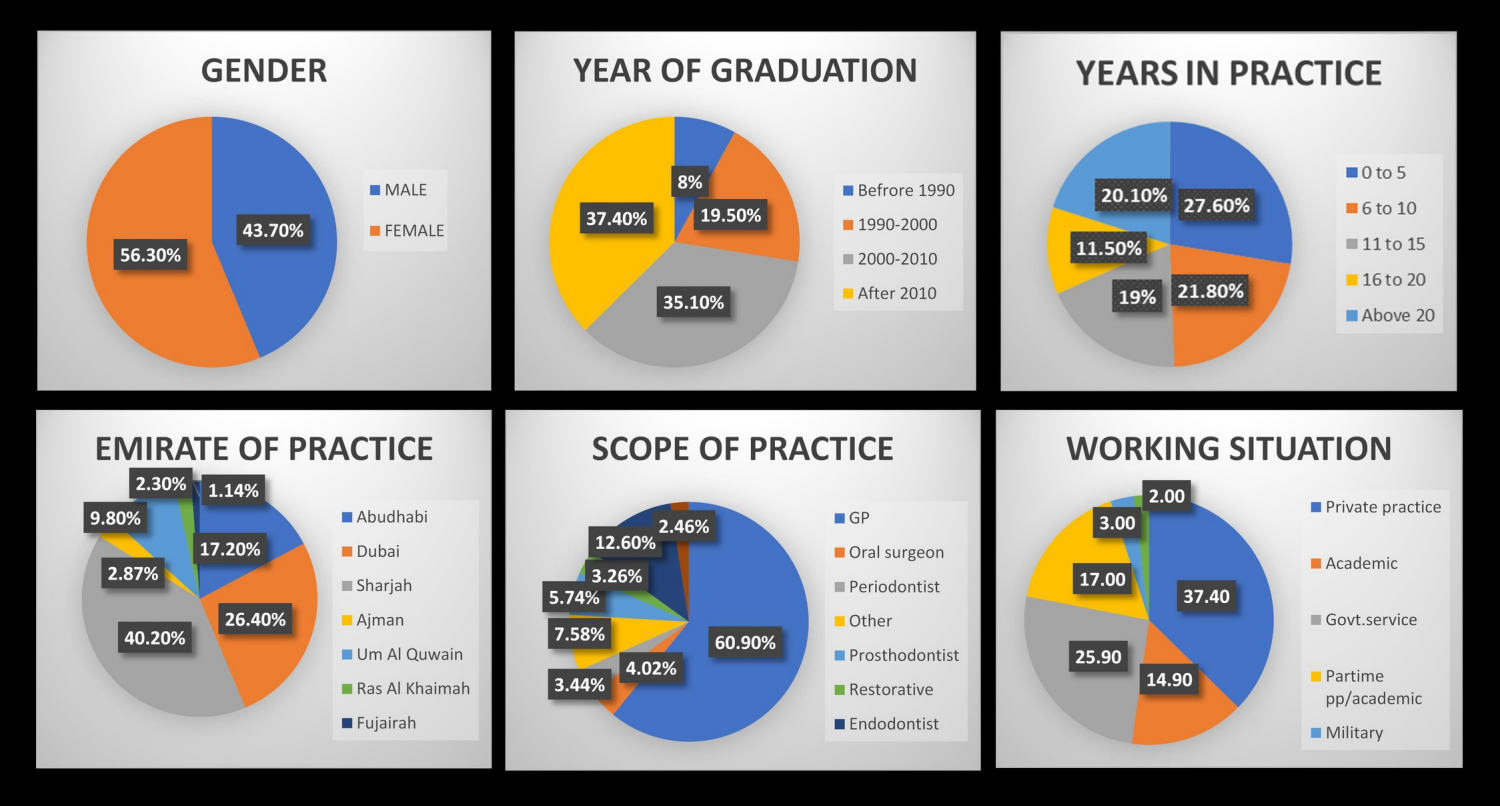
Socio-demographic characteristics of participants.

Out of 250 dentists invited to participate in the study, 174 completed the survey (70% response rate). The socio-demographic characteristics of the participants are presented in Fig 1. Of the 174 participants, 106 (60.9%) were GDPs and 22 (12.6%) were endodontists and the remaining 46 (24.3%) were other dental specialists. The majority of respondents had been in practice for over 5 years (72%). A third of the respondents (37.4%), were employed in private practices, a quarter (25.9%) in government services, 14.9% were academics and the remaining were part-time practitioners and part-time academics (22%).

In terms of the frequency of antibiotic prescription, 38.5% (n = 67) prescribed antibiotics at least once a month, 17.2% (n = 30) prescribed them more than three times a week. The remainder prescribed antibiotics either thrice a week (10.3%, n = 18), twice a week (7.2%, n = 13), or once a week (12.1%, n = 21) and once in two weeks (14.4%, n = 25)

The participants were grouped into three categories based on the scope of practice. Group 1 comprised GDPs, Group 2, Endodontists, and the Group 3 the remainder of the specialists. Table 1 shows the antibiotic prescribing trends as per the clinical scenarios by the latter three groups. It was noteworthy that all endodontists prescribed antibiotics for situations with necrotic pulp and acute apical abscess, swelling and moderate to severe pre-operative symptoms (p = 0.025), in comparison to GDPs (91.5%, n = 97) and other specialists (80%, n = 36).

**Table 1:** The respondents antibiotic prescribing trends in specific clinical situations.

		N	Antibiotic Prescription N (%)	No antibiotic Prescription N (%)	p-value
Clinical situations	Scope of Practice				
Irreversible pulpitis; mod/severe pre-op symptoms	Gen.Practitioner	106	13(12.3)	93(87.7)	0.88
	Endodontist	22	2(9.1)	20(91.0)	
	Others	45	6(13.3)	39(86.7)	
Irreversible pulpitis with symptomatic apical periodontitis; mod/severe pre-op symptoms	Gen.Practitioner	106	21(19.8)	85(82.0)	0.65
	Endodontist	22	6(27.3)	16(72.7)	
	Others	45	8(17.8)	37(82.2)	
Necrotic pulp with symptomatic apical periodontitis; no swelling, mod/severe pre-op symptoms	Gen.Practitioner	106	19(17.9)	87(82.1)	0.62
	Endodontist	22	5(22.7)	17(77.3)	
	Others	45	11(24.4)	34(75.6)	
Necrotic pulp with chronic apical abscess; sinus tract present; no/mild pre-op symptoms	Gen.Practitioner	106	24(22.6)	82(77.4)	0.58
	Endodontist	22	3(13.6)	19(86.4)	
	Others	45	11(24.4)	34(75.6)	
Necrotic pulp with chronic apical abscess; sinus tract present; moderate /severe pre-op symptoms	Gen.Practitioner	106	28(26.4)	78(73.6)	0.29
	Endodontist	22	5(22.7)	17(77.3)	
	Others	45	17(37.8)	28(62.2)	
Necrotic pulp with acute apical abscess; swelling present; mod/severe pre-op symptoms	Gen.Practitioner	106	97(91.5)	9 (8.5)	0.02*
	Endodontist	22	22(100)	0 (0.0)	
	Others	45	36(80.0)	9(20.0)	
Avulsion	Gen.Practitioner	106	30(28.3)	76(71.7)	0.51
	Endodontist	22	4(18.2)	18(81.8)	
	Others	45	10(22.2)	35(77.8)	
I&D of localized intraoral swelling, no extra-oral swelling	Gen.Practitioner	106	32(30.2)	74(69.8)	0.89
	Endodontist	22	6(27.3)	16(72.7)	
	Others	45	12(26.7)	33(73.3)	
I &D of diffuse intraoral swelling,no external swelling present	Gen.Practitioner	106	62(58.4)	44(41.5)	0.91
	Endodontist	22	12(54.5)	10(45.5)	
	Others	45	27(60.0)	18(40.0)	
I&D of diffuse intraoral swelling, external swelling present	Gen.Practitioner	106	90(84.9)	16(15.1)	0.41
	Endodontist	22	21(95.5)	1(4.5)	
	Others	45	39(86.7)	6(13.3)	
Post-operative pain after instrumentation or obturation	Gen.Practitioner	106	7(6.6)	98(92.5)	0.19
	Endodontist	22	5(22.7)	17(77.3)	
	Others	45	5(11.2)	40(88.9)	
Retreatment of gutta-percha/silver point	Gen.Practitioner	106	5(4.7)	101(95.3)	0.05*
	Endodontist	22	4(18.2)	18(81.8)	
	Others	45	2(4.4)	43(95.6)	
Perforation repair (before or after)	Gen.Practitioner	106	7(6.6)	99(93.4)	0.27
	Endodontist	22	0(0.0)	22(100.0)	
	Others	45	1(2.2)	44(97.8)	
Endodontic surgeries (before or after)	Gen.Practitioner	106	40(37.7)	66(62.3)	0.17
	Endodontist	22	13(59.1)	9(40.9)	
	Others	45	20(44.4)	25(55.6)	

Chi-square test

* represent significant at p-value (p<0.05)

The most popular antibiotic prescribed by the respondents (in non-allergic patients) was 500 mg amoxicillin thrice daily (n = 76, 43.7%), followed by 500 mg of metronidazole thrice a day (n = 65, 37.4%). Amoxicillin with clavulanic acid was also prescribed for endodontic infections (n = 37, 21.3%). Penicillin VK 500 mg was prescribed by only a very small minority, 3.4% (n = 6) of the respondents.

The antibiotic of choice for penicillin allergic patients was erythromycin 500 mg, thrice daily and was prescribed by a fifth of the respondents (n = 37, 21.3%), followed by clindamycin 300 mg twice daily which was prescribed by 14.4% (n = 25) of the respondents. Around 9.8% (n = 17) of the respondents reported using azithromycin (500 mg once daily for a 3days course) and 7.5% (n = 13) respondents preferred to use cephalexin 500 mg twice daily for 5days. Above 75% of respondents(n = 130) in our survey prescribed a minimum 5-day dosage for an antibiotic course. Those who prescribed a seven-day course were 20% (n = 35) while a few (5%, n = 9) prescribed a three-day course.

On further questioning the antibiotic prescribing practices of the respondents, a loading dose, in cases of infection, was prescribed by a fifth (21.3%; n = 37) and the majority appeared not to prescribe a loading dose. A small minority (15%; n = 26) of the respondents prescribed antibiotics ahead of weekends or upcoming holidays, anticipating that the clinician/s may not be accessible to their patients during an intervening putative dental emergency. If the prescribed antibiotic was ineffective after 2–3 days, 40.2% of the respondents preferred to add a second antibiotic. Interestingly, 22.4% of the dentists opted to extend the duration of the antibiotic if the prescribed drug was ineffective.

When questioned on guidelines followed by dentists for antibiotic prescription, 58% (n = 101) followed local guidelines, and 42% (n = 73) did not follow any specific guideline *per se*. Of the 101 dentists who followed guidelines, 52% (n = 53) followed the antibiotic prescribing guidelines promulgated by the Ministry of Health, UAE, while 22% (n = 23) followed the American Association of Endodontists guidelines. Another 22% (n = 23) followed their own institutional guidelines. The remaining 4% (n = 4) followed the guidelines set by the ESE (European Society for Endodontology) and ASE (Australian Society of Endodontology), respectively.

The association between the frequency of antibiotic prescription and other variables is shown in Table 2. Interestingly, there was a significant association between the frequency of antibiotic prescription and the number of years in practice (p = 0.024), indicating that the long serving practitioners tended to prescribe antibiotics more frequently than those who were in practice for a shorter period of time. There was also a significant association between the frequency of antibiotic prescription and gender (p = 0.049), where female dentists prescribed antibiotics more frequently than their male counterparts. However, there was no association between the frequency of antibiotic prescription and the scope of practice (p = 0.377).

**Table 2:** Data on the frequency of antibiotic prescription by the survey participants.

Variables	Frequency of antibiotic prescription			
	>3x/wk n (%)	1-3x/wk n (%)	once/2wks-once/mth n (%)	p- value*
**Gender**				
Male	19 (63.3)	12 (38.7)	44 (38.9)	0.049*
Female	36.7 (11.1)	19 (61.3)	69 (61.1)	
**Scope of practice**				
Gen. Practitioner	16 (15.1)	20 (18.9)	70 (66.0)	0.377
Endodontists	11 (24.2)	5 (11.1)	29 (64.4)	
Others	3 (13.6)	6 (27.3)	13 (59.1)	
**Years in practice**				
0–5	3 (10.0)	7 (22.6)	39 (34.5)	0.024*
6–10	7 (23.3)	4 (12.9)	27 (23.9)	
11–15	4 (13.3)	8 (25.8)	21 (18.6)	
16–20	5 (16.7)	6 (19.4)	8 (7.1)	
>20	11 (36.7)	6 (19.4)	18 (15.9)	

Chi-square test

* represent significant at p-value (p<0.05)

## Discussion

The response rate for this web-based survey, using a Google Response form, was 70%, a rate considered satisfactory for on-line surveys [19]. We used a convenience sampling method where members of the EDS (Emirates Dental Society) were invited to participate in the study. A similar online survey by Germack et al, [17] done in USA had a response rate of only 22.86%. It is possible that our response rate was much higher because of the sampling method employed. Our goal, nevertheless, was not to obtain a percentage of dentists relative to the size of the population. Navabizadeh et al., (2011) in Iran also used questionnaires to assess antibiotic prescriptions for endodontic infection and reported a response rate of 46.5% [20]. Similarly, a study by Maslamani and Sedeqi (2018) determined prescribing patterns of antibiotics and analgesics among dentists for endodontic infection using questionnaires and reported a response rate of 75.6% [21] similar to the current study. Al Khabuli et al., (2016) investigated the knowledge and attitude of dentists to antibiotic prescription in the Northern Emirates of the UAE reporting a response rate of 77% [16].

In our survey, the respondents were presented with commonly seen pulpal and periapical clinical scenarios and were asked whether they would prescribe an antibiotic in such situations. As medical history and specific details of the symptoms in each scenario could not be included, due to the nature of the study, the current data must be interpreted with caution. Accordingly, 89% of the respondents prescribed antibiotics for necrotic pulp with acute apical abscess with swelling and moderate to severe pre-operative symptoms. Similar responses of 87% and 99% have been recorded in two previous studies in USA and Saudi Arabia, respectively for very similar clinical scenarios [17, 21]. This clinical scenario is generally considered by authorities as an indication for antibiotic prescription combined with debridement of the root canal and/or incision and drainage [18]. Furthermore, significant difference in the prescription pattern between GDPs and endodontists for this condition was noted in our study (p<0.05). Thus, all endodontists prescribed antibiotics for necrotic pulp with acute apical abscess with swelling and moderate to severe pre-operative symptoms, whereas only 80% of other specialists and 91.5% of GDPs prescribed antibiotics. One reason for this could be the poor understanding of the disease process and its management rationale.

It was also disconcerting to note that a good proportion of respondents unnecessarily prescribed antibiotics for patients with irreversible pulpitis (12%), necrotic pulps with no systemic involvement (20%) as well as for cases of necrotic pulps with sinus tracts (21%). Non-surgical root canal therapy without antibiotics is usually the standard treatment for such cases according to AAE guidelines [1], and analgesics, not antibiotics, are indicated for pain from pulpitis or periapical inflammation according to the latter authorities. In a Brazilian survey by Bolfoni et al. [22], the frequency of antibiotic prescription for the necrotic pulps with no systemic involvement scenario was even higher with 88.1% of the respondents prescribing antibiotics. The decision on whether to prescribe an antibiotic or not in specific clinical situations should be evidence based [23–27]. Our data reveal that indiscriminate antibiotic prescription by general practitioners in UAE, which appear to be not uncommon, needs to be addressed both, by continuing professional education courses, and also emphasizing the importance and need to follow the antibiotics prescribing guidelines promulgated either by local or international professional bodies.

It was comforting to note that prescription profile of antibiotics for periapical infections in our study was similar to that of a North American study by Germack et al. [17], except in the case of tooth avulsion where an overwhelming proportion (70%) of US dentists prescribed an antibiotic. In our study, less than a third (29%) of respondents prescribed an antibiotic in the case of an avulsed tooth. Although there is some controversy over the use of systemic antibiotics in the latter situation, the International Association of Dental Traumatology recommends antibiotic prescription based on evidence from experimental data [28].

There was also a significant difference in the prescribing pattern between GDP’s, endodontists, and other specialists in retreatment cases (p = 0.05) where endodontists prescribed antibiotics more frequently (18.2%), compared with GDPs (4.7%) and other specialists (4.4%). One possible explanation for this practice could be that retreatment procedures are generally chronic, intractable cases, that are often referred to endodontists. Moreover, the AAE recommends prescribing antibiotics in persistent, chronic infections with exudates which are not resolved by intracanal procedures and antiseptics alone [1].

The antibiotics of choice, preferred by respondents were similar to those chosen by counterparts in the region, in such as Iran, Kuwait and Saudi Arabia [20, 21, 29]. Thus, for a majority of respondents (43.7%), amoxicillin was the antibiotic of choice for endodontic infections. Amoxicillin is a moderate-spectrum, bacteriolytic, beta-lactam antibiotic [30]. On the other hand, an amoxicillin+clavulanic acid combination, with a wider spectrum, was also prescribed by a smaller proportion of respondents as the drug of first choice in endodontic infections. Penicillin VK was prescribed by only 3.4% of the respondents in our study. An antibiotic survey amongst members of the AAE in 2002 [18] revealed that, penicillin VK was the principal antibiotic prescribed by dentists (61.48%), and amoxicillin was prescribed only by 27.5%. However, a more recent survey, in 2016, of the AAE members showed that amoxicillin was currently more often prescribed (60.71%), followed by penicillin VK (30.43%) [17]. The advantages of amoxicillin over penicillin are better absorption, longer shelf-life and sustained serum levels that permits a thrice daily dosage regimen compared to four times, daily, for penicillin VK [31].

It is generally accepted that clindamycin is the preferred alternative for penicillin-allergic patients [18, 20, 32], and yet only a small proportion of our respondents (14.4%) followed this recommendation, as a significant proportion preferred to prescribe erythromycin instead (21.3%). The latter is a macrolide, with a spectrum of activity similar to penicillin, whilst clindamycin, is a broader-spectrum antibiotic but narrow in its specificity and is particularly active against oral pathogens [33]. Azithromycin was used by 9.8% of respondents in cases of penicillin allergies. It is a semi-synthetic derivative of erythromycin which has a broader spectrum of antibacterial activity than the latter and better tissue penetration [34].

Over a third of our respondents (37.4%) prescribed metronidazole as a primary antibiotic for dental infections. Metronidazole is effective against obligate, but not facultative anaerobes, and has to be used in combination with other agents to obtain resolution of mixed aerobic-anaerobic, oral infections [35]. However, if the initial antibiotic was ineffective after 2–3 days, 40.2% of the dentists preferred to add metronidazole as a supporting antibiotic.

Regarding the duration of antibiotic dosage, the majority of the dentists prescribed a five-day course for most antibiotics, which is the generally accepted guideline for oro-facial infections [36]. This finding is similar to previous studies where a minimum five-day course was prescribed [6, 37]. Endodontic infections typically have a rapid onset and short duration, of 2 to 7 days or less, particularly if the infective cause is eliminated [38]. It is also interesting to note that 22.4% of the dentists opted to extend the duration of the antibiotic if it was not effective. It is noteworthy, that the prolonged use of antibiotics or an ineffective dose contribute to the emergence of microbial resistance [18].

In this survey, only 21.2% of dentists prescribed a loading dose in case of infections. Literature shows that an antibiotic loading dose should be used whenever the half-life of the antibiotic is longer than 3 hours or whenever a delay of 12 hours or more is unacceptable to achieve therapeutic blood levels [14]. Most antibiotics used for orofacial infections have half-lives of less than 3 hours, but the acute nature of these infections require high therapeutic blood levels [38]. Therefore, a loading dose may ensure rapid elevation of therapeutic blood levels of the antibiotic to help combat the infection efficiently.

In our study, a small number of dentists (15%), prescribed antibiotics in case they were not accessible to their patients because of an approaching weekend/travel plan. The commonest reason given was to control infections in case of development of a swelling over the weekend and some practitioners preferred that a patient should be under antibiotic cover before any invasive procedure was done. The decision to prescribe antibiotics should not be influenced by patient demand, expectation of referring dentists, or if the patient has no access to a dentist before a weekend or holiday. Previously, it was shown that many endodontists felt compelled to prescribe antibiotics for every endodontic case to satisfy both the patients and the referring general practitioners demands [18]. These are reasons which constitute inappropriate antibiotic use.

There was a significant association between the frequency of antibiotic prescriptions and the number of years in practice (p<0.05). Respondents who were in practice for longer period were likely to prescribe antibiotics more frequently compared with those with a lesser period in practice. This could be due to the younger dentists already being trained or being aware of the emergence of antibiotic resistance and antibiotic abuse.

There was also a significant association between frequency of antibiotic prescription and gender (p < 0.05), where female dentists prescribed antibiotics more frequently compared to male dentists. Similar findings were seen in the study by Bjelovucic et al., (2019) where female dentists prescribed more antibiotics compared to male dentists [39]. We are unable to offer any specific reason for this rather unusual finding, but we concur that antibiotic prescriptions could be influenced by unspecified factors.

A limitation of our study was the use of convenience sampling technique. Accordingly, the findings of this study should be interpreted with caution, given that the selected sample was randomly chosen from all dentists who are registered in different Emirates. Although the response rate in this study is relatively high (70%), it is possible that dentists who agreed to participate in this survey are different in their knowledge of antibiotics prescription, compared to those who did not respond to the online questionnaire. In addition, the results of the survey showed a larger participation from younger dentists when compared to the older dentists and could be speculated that younger dentists were more responsive through electronic media.

### Conclusions

In general, the antibiotic prescribing practices of UAE cohort of dentists responding to our questionnaire appear to be congruent with the international norms, despite the fact that a surprising number did not follow specific antibiotic guidelines. There were, however, instances of inappropriate antibiotic use for specific endodontic pathologies. Dentists need to realize that most endodontic infections can be resolved by operative interventions alone without antibiotics. Our findings support the need for implementation of strategies to reduce the prescription of antibiotics for endodontic infections, continuing dental education courses, as well as adherence to regional guidelines which are important to raise the awareness of good antibiotic prescribing practices amongst dentists in UAE, in order to suppress the emergence of antibiotic resistance organisms.

## Supporting information

S1 Appendix The survey questionnaire used in the study. (DOCX)
