# Supplementary material for: Antibiotic prescribing practices of dentists for endodontic infections; a cross-sectional study
Source: PLoS One. 2020 Dec 30;15(12):e0244585. doi: 10.1371/journal.pone.0244585 (PMC7773263; doi:10.1371/journal.pone.0244585)
Supplement: S1 Appendix — (DOCX) [file pone.0244585.s001.docx]

# Supplimentary information: The survey questionnaire

| **Baseline demographics** | | | | |
| --- | --- | --- | --- | --- |
| **1**.**What year did you graduate from dental school?**  Before 1990 ▢ 1990-2000 ▢  2000-2010 ▢ After 2010 ▢ | | | **7. On average, based on cases you see, how many times in a week do you prescribe antibiotics?**  once a month ▢ once in 2 weeks ▢  once a week ▢ twice a week ▢  thrice a week ▢ more than three times ▢ | |
| **2.Scope of practice**  GDP ▢ Implant ▢  OS ▢ Pros ▢  Perio ▢ Endo ▢  Restorative ▢ Ortho ▢  Community ▢ Others ▢ | | | **8. In which of the following situations would you prescribe antibiotics? (Please select all that apply)**  ▢ irreversible Pulpitis; mod/severe pre-op symptoms.  ▢ Irreversible Pulpitis with symptomatic apical periodontitis;  mod/severe pre-op symptoms  ▢ Necrotic Pulp with Symptomatic Apical Periodontitis; no  swelling, mod/severe pre-op symptoms  ▢ Necrotic Pulp with Chronic Apical Abscess; sinus tract  present; no/mild pre-op symptoms  ▢ Necrotic Pulp with Chronic Apical Abscess; sinus tract  present; mod/severe pre-op symptoms  ▢ Necrotic Pulp with acute apical Abscess; swelling Present;  mod/severe pre-op symptoms | |
| **3. Gender**  Male ▢ Female ▢ | | |  |  |
| **4. Years in practice**  0-5 ▢ 5-10 ▢ 10-15 ▢ 15-20 ▢ above 20 ▢ | | |  |  |
| **5.Which emirate do you practice at?**  Abudhabi ▢ Al Ain ▢  Dubai ▢ Sharjah ▢  Ajman ▢ Um Al Quwain ▢  Fujairah ▢  RasAlKhaimah ▢ | | |  |  |
|  |  |  | **9. In which of the following situations would prescribe antibiotics? (please select all that apply)**  ▢ Avulsion  ▢ I & D of a localized intraoral swelling, no external swelling  ▢ I & D of a diffuse intraoral swelling, no external swelling  ▢ I & D of a diffuse intraoral oral swelling + external swelling present  ▢Post-op pain after instrumentation or obturation  ▢ Retreatment of gutta percha/silver point  ▢ Perforation repair (before or after)  ▢ Endodontic surgeries (before or after) | |
| **6. Which of the following best describes your working situation**  Private practice (PP) ▢ Academics ▢  Government service ▢ PP+ Academics ▢  Part-time PP ▢ Military ▢ | | |  |  |
| **Antibiotic selection** | | | | |
| **10.Please select the antibiotic and dosage you prescribe most often for patients with no medical allergies.** | | | | |
| **Drug** | **Dosage** | | | **Number of days ( 3 days, 5 days, 7 days)** |
| Amoxicillin |  | | |  |
| Ampicillin |  | | |  |
| Augmentin |  | | |  |
| Azithromycin (Zithromax) |  | | |  |
| Cephelexin |  | | |  |
| Clindamycin |  | | |  |
| Erythromycin |  | | |  |
| Metronidazole |  | | |  |
| Penicillin V |  | | |  |
| Others | Dosage | | | Number of days: |
| **11. Please select the antibiotic and dosage you prescribe most often for patients with an allergy to penicillin.** | | | | |
| **Drug** | **Dosage** | | | **Number of days (3 days, 5 days, 7 days)** |
| Azithromycin |  | | |  |
| Cephalexin |  | | |  |
| Clindamycin |  | | |  |
| Erythromycin Base |  | | |  |
| Metronidazole |  | | |  |
| Other: | Dosage | | | Number of days |
| **Antibiotic prescribing practices** | | | | |
| **12. Do you prescribe a loading dose**  Yes ▢ No ▢ | | **15. Do you ever prescribe antibiotics to your patients if you are not accessible due to an upcoming weekend/holiday**  Yes ▢ No ▢  **15 A. If yes, please explain**  _________________________________ | | |
| **13.If your antibiotic prescription is ineffective after 2-3 days what would you do?**  A. Change antibiotics?   - B. Add a second antibiotic?   C. Extend the duration of the current antibiotic | | **16. Do you have guidelines for antibiotic use at your practice/hospital/university?**  Yes ▢ No ▢  **16 A. If yes, please tick which**  Institutional ▢  MOH (Ministry of Health) ▢  AAE (American Association of Endodontists) ▢  ESE (European Society of Endodontology) ▢  ASE (Australian Society of Endodontology) ▢ | | |
| **14. If you decide to change, to which antibiotic**  **Short answer text**  _____________________________________________ | |  |  |  |
